# CircRNA identification and feature interpretability analysis

**DOI:** 10.1186/s12915-023-01804-x

**Published:** 2024-02-27

**Authors:** Mengting Niu, Chunyu Wang, Yaojia Chen, Quan Zou, Ren Qi, Lei Xu

**Affiliations:** 1 School of Electronic and Communication Engineering, Shenzhen Polytechnic University, Shenzhen, 518055 China; 2 Postdoctoral Innovation Practice Base, Shenzhen Polytechnic University, Shenzhen, 518055 China; 3 School of Life Science and Technology, University of Electronic Science and Technology of China, Chengdu, China; 4 Faculty of Computing, Harbin Institute of Technology, Harbin, 150000 Heilongjiang China; 5 Institute of Fundamental and Frontier Sciences, University of Electronic Science and Technology of China, No.4 Block 2 North Jianshe Road, Chengdu, 610054 China; 6 Yangtze Delta Region Institute (Quzhou), University of Electronic Science and Technology of China, Quzhou, China

**Keywords:** CircRNA, Feature, Deep learning, Interpretation, SHAP

## Abstract

**Background:**

Circular RNAs (circRNAs) can regulate microRNA activity and are related to various diseases, such as cancer. Functional research on circRNAs is the focus of scientific research. Accurate identification of circRNAs is important for gaining insight into their functions. Although several circRNA prediction models have been developed, their prediction accuracy is still unsatisfactory. Therefore, providing a more accurate computational framework to predict circRNAs and analyse their looping characteristics is crucial for systematic annotation.

**Results:**

We developed a novel framework, CircDC, for classifying circRNAs from other lncRNAs. CircDC uses four different feature encoding schemes and adopts a multilayer convolutional neural network and bidirectional long short-term memory network to learn high-order feature representation and make circRNA predictions. The results demonstrate that the proposed CircDC model is more accurate than existing models. In addition, an interpretable analysis of the features affecting the model is performed, and the computational framework is applied to the extended application of circRNA identification.

**Conclusions:**

CircDC is suitable for the prediction of circRNA. The identification of circRNA helps to understand and delve into the related biological processes and functions. Feature importance analysis increases model interpretability and uncovers significant biological properties. The relevant code and data in this article can be accessed for free at https://github.com/nmt315320/CircDC.git.

**Supplementary Information:**

The online version contains supplementary material available at 10.1186/s12915-023-01804-x.

## Background

In eukaryotes, pre-mRNAs are backspliced to produce noncoding RNAs with covalently bonded circular structures named circular RNAs (circRNAs) [1]. Unlike linear RNA, circDNA has a closed circular structure, is not affected by exonucleases, and has a more stable expression [2]. CircRNA, which subverts the central dogma of classical gene expression theory, has gradually become popular in noncoding RNA research [3]. With the help of RNA-seq technology, a large number of algorithms for identifying circRNAs have emerged [4–6]. Examples include Tophat-fusion [7], Mapsplice [8], and segemehl [9]. To date, more than 100,000 kinds of circRNAs have been found in many species [10]. CircRNA has coding function, is an important participant in the development of diseases [11, 12], is a steady-state product of mRNA splicing, participates in complex gene expression regulation in a new way, and has important noncoding functions [13, 14]. CircRNAs contain a large number of miRNA binding sites and function as miRNA sponges. By inhibiting miRNA, circRNA indirectly regulates the expression of mRNA, participates in the occurrence and development of many human tumours, and may become a new marker [15]. The detection of circRNAs is critical to understanding their biogenesis and purpose.

When performing circRNA verification, biotechnology such as chips and qRT‒PCR is needed [16]. Using biological experimental methods to identify circRNAs and discover the relationship between circRNAs and diseases is time-consuming and laborious. It is particularly important to develop bioinformatics methods to efficiently identify circRNAs. With the accumulation of circRNA sequence data and the development and maturity of supervised classification algorithms, researchers have focused on applying machine learning (ML) to circRNA identification to improve the efficiency and accuracy of circRNA identification. Pan et al. extracted sequence features, used random forest (RF) to identify circRNAs, and built the WebCircRNA server [17]. Pan proposed a multikernel learning model named PredcircRNA based on multiple functional training to classify circRNA and lncRNA [18]. Chen proposed a hierarchical algorithm named H-ELM with feature selection based on the extreme learning machine(ELM) algorithm to classify circRNAs [19]. In our previous work, we proposed to use ELM improved by particle swarm optimization for circRNA identification [20]. Mohamed Chaabane built a deep learning(DL) framework named circDeep to improve the classification of circRNAs by learning feature representations of different modalities [21]. PCirc uses an RF to identify plant circRNAs based on the composition characteristics of plant circRNA coding sequences [22]. Most of the existing circRNA identification algorithms directly use manual features when characterizing sequence features and seldom consider the factors affecting circRNA loop formation. Therefore, it is worthwhile to fully analyse and encode the looping characteristics of circRNAs and use ML [23, 24] to achieve more accurate identification.

**Fig. 1:**
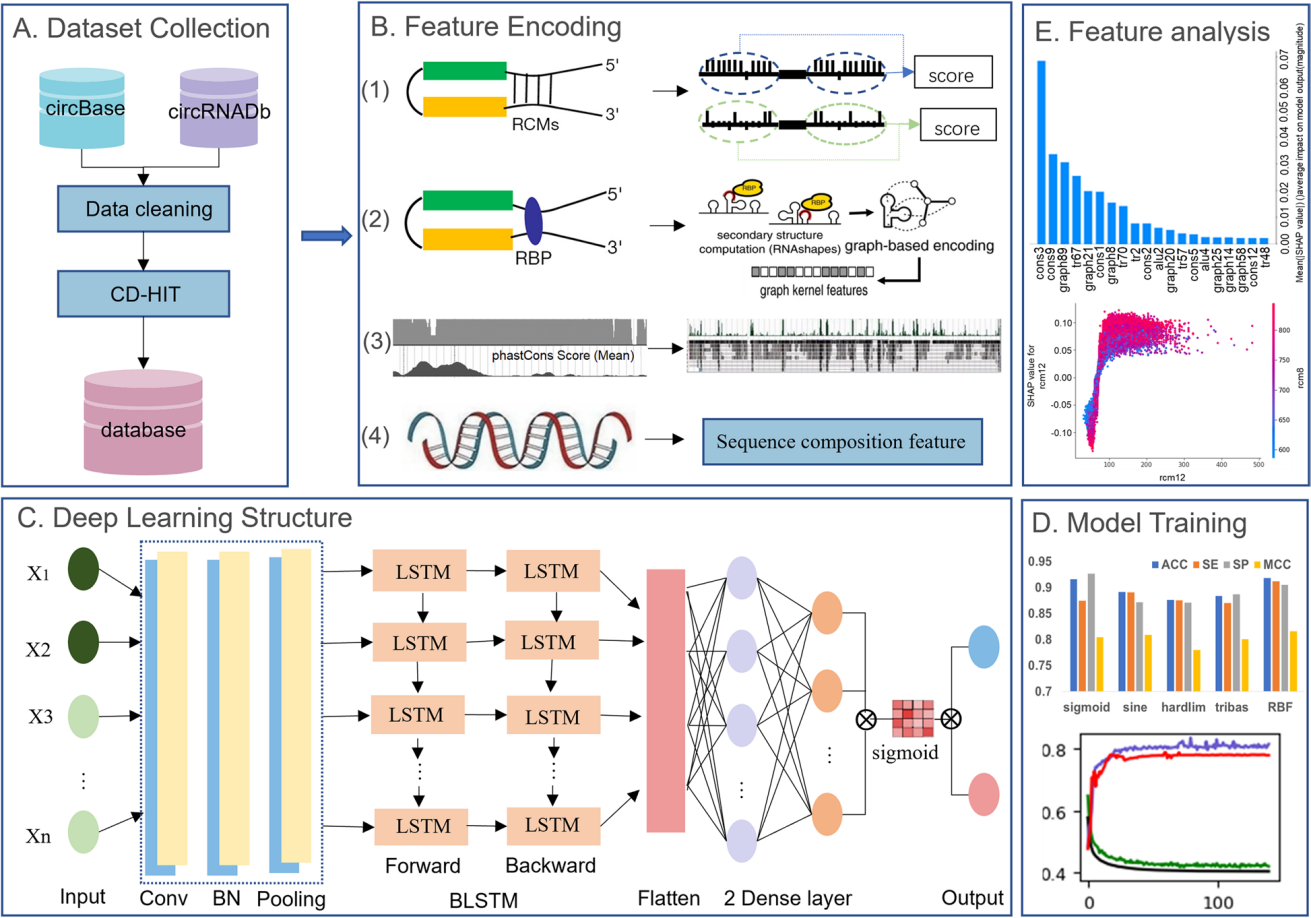
Structure and functionality of the online portal CircDC. **A** Dataset collection. **B** Feature encoding. **C** Deep learning structure. The structure of the CircDC, including the input layer, convolutional layers, merger layers, inception module, attention layers, fully connected layers and output layer. **D** Model training. **E** Feature analysis

We developed a new tool, CircDC, for circRNA prediction. First, the reverse complementarity matching features (RCMs), conservative score, graph structure and sequence composition were constructed according to the factors affecting the loop formation of circRNA. Important feature selection is then performed using the max-relevance-max-distance (MRMD) v2 method [25]. A convolutional neural network (CNN) and bi-directional long short-term memory (BLSTM) network are used for deep feature learning and classification. The classification accuracy, robustness and scalability of the CircDC model are proven through tenfold cross-validation (TFCV) and independent test set validation, and the important features affecting the model are analysed through interpretability features. The CircDC frame structure is shown in Fig. 1.

## Results

### Model design and training

**Fig. 2:**
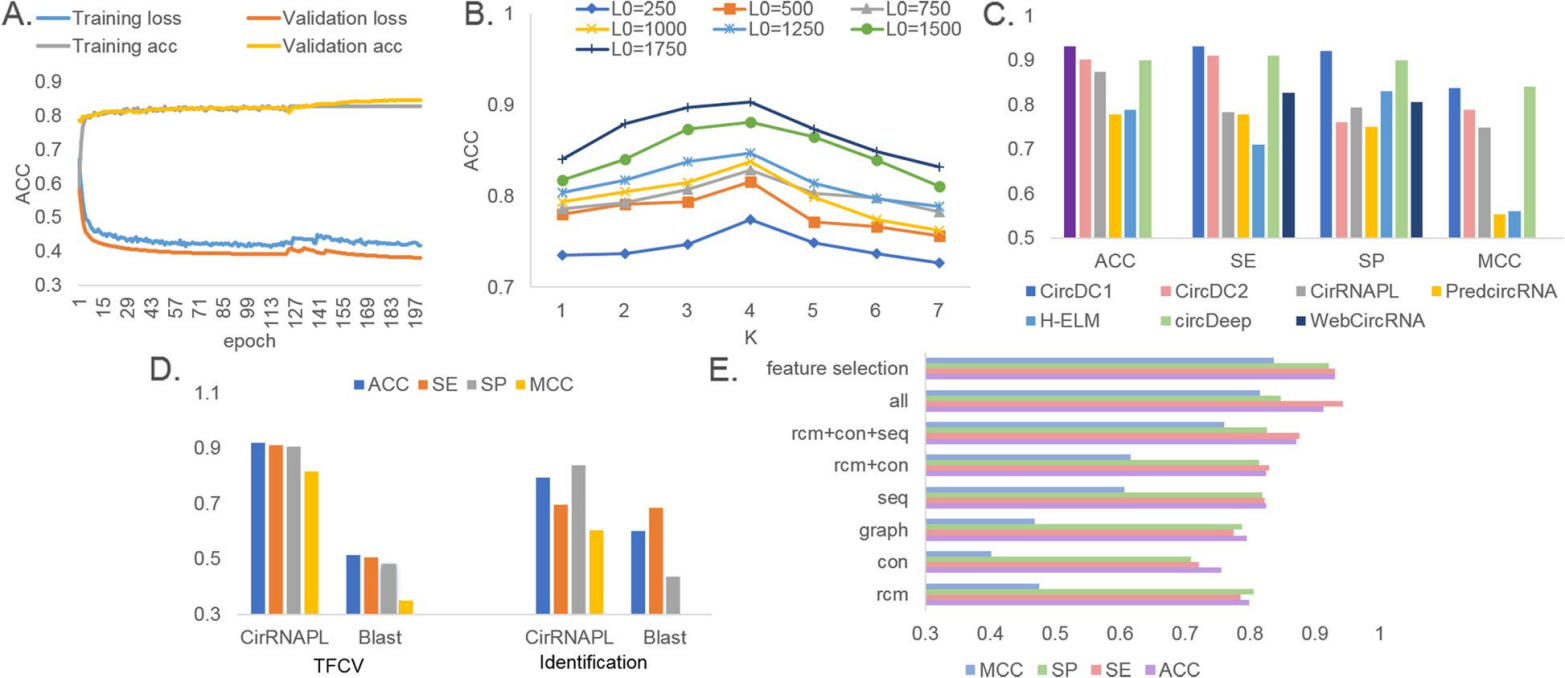
**A** Model performance analysis under different EPOCH. **B** k and L parameter optimization. **C** Performance comparison with state-of-the-art methods. **D** Performance comparison with Blast. **E** Performance comparison of feature combination strategies

The first step includes model design and training. To obtain a highly robust DL model, the first key issue is hyperparameter optimization. Model training and selection is a challenging step in DL. The configuration and selection of parameters are crucial for DL models. Based on TFCV, we used accuracy (ACC) to evaluate each parameter setting. The parameters for tuning include batch size, learning rate (LR), number of iteration epochs and maximum sequence length. In the training phase, to speed up the model training, the Adam gradient descent method is used to update the LR, with ACC as the objective function. When the ACC value reaches the maximum, the iteration is stopped, and the optimal parameters are saved. The initial value of the LR is 0.001. We used several combinations of hyperparameters to obtain the optimal combination. To reduce model overfitting, we also used a dropout rate for each model. The final optimal architecture and hyperparameters are shown in Additional file 1. Then we statistically analyze the changes in loss and ACC of the training set and validation set. Analyze the changes in model performance and model convergence under different epochs (Fig. 2A). It can be seen that as epoch increases, the training ACC and validation ACC of CircDC both show an upward trend, and the training loss and validation loss show a downward trend and gradually become stable. This change is in line with the trend of gradual optimization of model training, without overfitting. And when epoch is 123, the model gradually becomes stable. The training results are gradually optimized, and there is no overfitting. It proves that the model training is optimal.

When calculating the features of RCMs, there are two important parameters, k and L. Therefore, we optimized these two parameters. For the corpus optimization of k-mer sequences, we performed k-optimization through a grid search algorithm, where the step size was set to 1, to generate the corpus of k-mer sequences. The performance of the model under different k values was counted separately. L ∈ [250,1750], with a step size of 250. The result is shown Fig. 2B. For the k value, when k = 3, the performance of the model was optimal for all L; for L, as L increases, the ACC value also increased. However, the ACC values did not change for larger k-mers and longer flanking sequences. Therefore, the k-mer vocabulary was V = 4^3^ = 256. It was also found that when L > 250 bps, the performance of the model gradually increased. This reflects that the RCMs that affect the formation of circRNA hairpins are mainly located after 250 bp, and when L > 500 bp, it is beneficial to the recognition of circRNAs.

### The proposed CircDC outperforms state-of-the-art methods

To verify the predictive ability of the CircDC model proposed in this study, we compared it with currently existing methods. CirRNAPL [20], WebCircRNA [17], PredcircRNA [18], and H-ELM [19] use datasets from the circBase database, and circDeep [21] uses datasets from the circRNADb database. Therefore, we applied CircDC to these two databases with TFCV (the results with the circBase dataset are labelled as CircDC1, and those with the circRNADb dataset are labelled as CircDC2). The results of the comparison methods were obtained from the corresponding literature, among which only the sensitivity (SE) and sensitivity (SP) indicator results were provided for WebCircRNA. The TFCV results are shown in Fig. 2C.

First, the performance of the CircDC1 model is significantly higher than that of WebCircRNA in terms of the SE and SP indicators. Second, compared with CirRNAPL, PredcircRNA, and H-ELM, which use ML algorithms and sequence composition features, CircDC1 has obvious advantages. The differences in the prediction results also prove that the feature encoding used in this paper has obvious significance in improving the performance of the model, and it also proves that the effect of the DL model is better than that of the ML algorithm under certain circumstances. CircDC2 also outperforms circDeep using DL algorithms. circDeep uses an asymmetric CNN and a BLSTM, and the structures of the two models are not much different; the biggest difference is the feature encoding scheme. In this paper, we further discuss the performance of the model feature encoding input into the circDeep network model.

### Comparison of the proposed CircDC over conventional Blast methods

When faced with an unknown sequence, we usually choose to use Blast [26] for sequence alignment and identify homologous genes. In this section, the identification effects of CircDC and Blast are compared. Blast uses default parameters. After applying Blast to a certain sequence A, the comparison result, including the identify, e-value, and query sequence, is obtained, and the value of the identify/sequence length is calculated. The results are arranged in descending order. The category of the sequence corresponding to the largest ratio is the category of sequence A. The experimental results of TFCV and independent test set validation are shown in Fig. 2D.

It can be seen that the predicted ACC of Blast is significantly lower than that of CircDC. The ACC of TFCV and Blast with the independent test set are 0.439 and 0.605, respectively, and the ACC of CircDC are 0.9305 and 0.8314, respectively. Since Blast only compares certain more or less important keywords in a sequence, it is not surprising that its accuracy is slightly lower. CircDC classification methods based on sequence data will have increasingly wide validity and usability in research.

### Exploration of the optimal feature combination for CircDC

To explore the optimal feature combination for CircDC and show whether the features constructed in this paper can explain the performance, we tested the performance of CircDC with different categories of features, single features and different feature combinations. The results of TFCV are shown in Fig. 2E.

The performance of the constituent feature sequence descriptor is higher than that of the other three descriptors. Among them, the ACC value is improved by more than 10% compared to that of the worst-performing RCMs descriptor, which reflects the enormous importance of sequence composition features to our task. Although the RCMs descriptor has the lowest performance, it appears to be effective in improving the performance when combined with other descriptors. As expected, that multifeature fusion yields higher performance than any single descriptor. This indicates strong complementarity among the three descriptors. In addition, to avoid deviations between different dimensionality reduction methods, we use the MRMD 2.0 algorithm, which combines multiple feature selection algorithms and improves the selection results based on the ranking algorithm. The performance after feature selection is also improved compared to the performance with all feature fusion, with an ACC value of 0.9305, which is better than that with all single features and with other multifeature fusions. This indicates that different feature information is complementary in the fusion strategy, and optimal features can capture more discriminative and high-quality features, thus effectively improving the predictive performance of the CircDC model.

### Exploration of the optimal structure for constructing CircDC

**Fig. 3:**
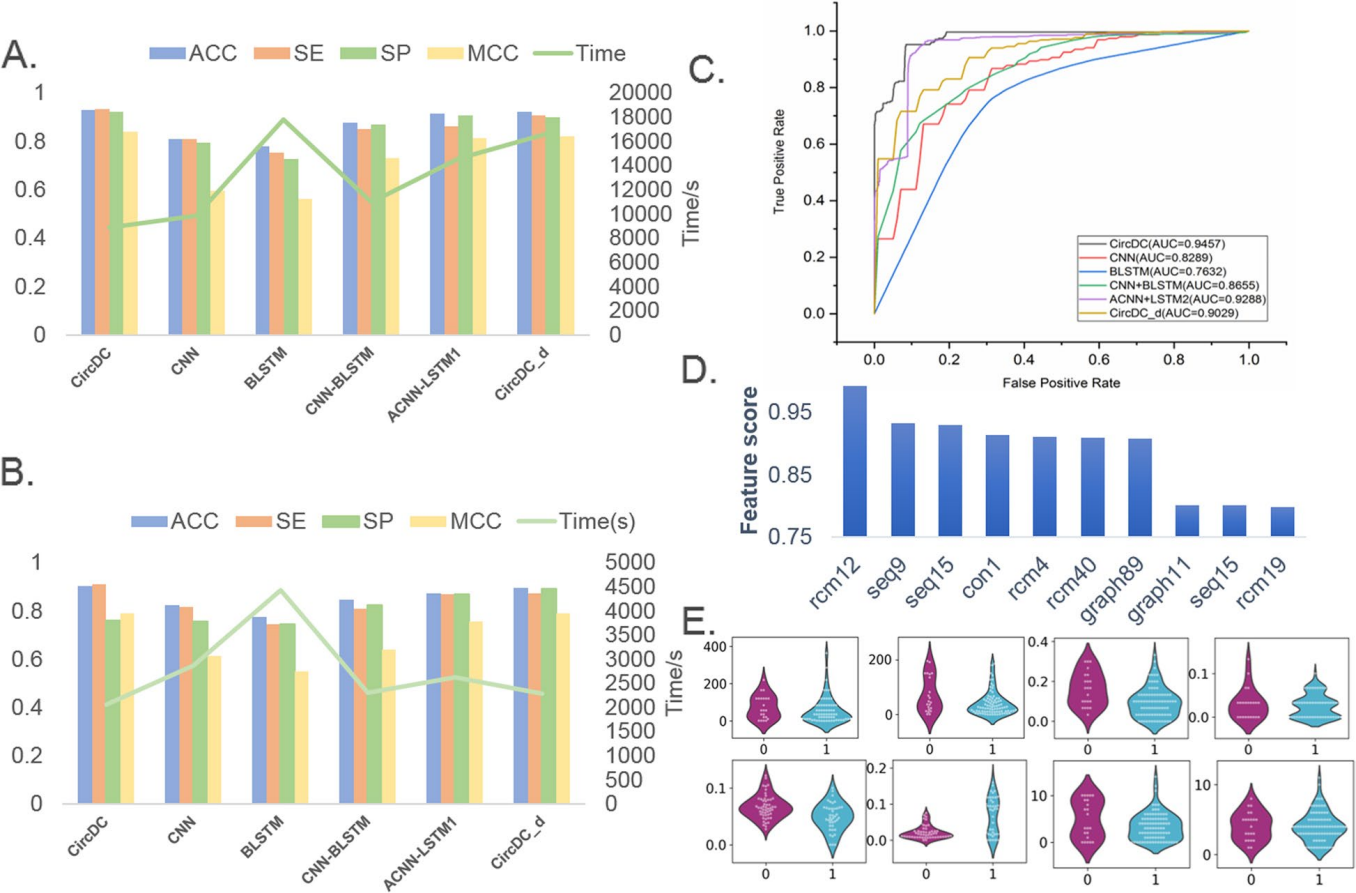
**A** Performance comparison of TFCV with different structures. **B** Performance comparison of independent test sets with different structures. **C** ROC curves of different structures. **D** Top 20 feature analysis. **E** Violin plot to visualize feature distribution

To explore the optimal structure for constructing CircDC, we compared the performance of the proposed architecture with that of several sequence classification benchmark frameworks. We compared the CNN architecture under one-hot encoding, BLSTM and CNN-BLSTM, the ACNN-LSTM combination (ACNN-LSTM1) under the encoding scheme in this paper, and the depth model (CircDC_d) in this paper under circDeep feature encoding. Among them, the frameworks of CNN, BLSTM, CNN-BLSTM, and ACNN-LSTM1 adopt the same parameter optimization algorithm as CircDC. More detailed parameter settings is in Additional file 1. The performance and running time of each framework were calculated. Figure 3A and B show the performance of TFCV and independent test set validation for all the above methods. Figure 3C shows the receiver operating characteristic curve (ROC) curve and AUC of TFCV.

First, it can be observed that the results of the CNN are higher than those of the BLSTM model, and the results of the CNN-BLSTM are significantly improved compared with those of the separate CNN and BLSTM, which indicates the importance of convolution in predicting circRNA tasks. The ACNN-LSTM1 framework achieved an ACC of 0.9149, which proves that the feature encoding scheme proposed in this paper includes richer feature information compared with the results of circDeep. The ACC of CircDC_d is 0.9215, which proves the effectiveness of the DL framework in this paper compared with the effectiveness of circDeep. CircDC’s ROC is also more stable than that of other structures, and its AUC value is also better than that of other comparison frameworks, proving its effectiveness. Through the polyline comparison of the running time, it can be found that the time of CircDC is also relatively small. The performance and operating efficiency of CircDC are demonstrated through experiments.

This also motivates further analysis of which features our feature encodes are most important to the classifier. After feature selection using the MRMD v2 method, feature score ranking results were output. Then, the top 10 features were analysed according to the output feature scores, and the feature names and feature scores were counted. Among them, the RCMs feature is recorded as the “rcm” + feature dimension, the conservative score is recorded as the “con” + feature dimension, the sequence composition feature is recorded as the “seq” + feature dimension, and the graph structure feature is recorded as the “graph” + feature dimension, as shown in Fig. 3D. Among the top 10 features are 4 RCMs features, 3 sequence composition features, 2 graph structure features, and 1 conservative score feature. Consistent with the performance observations, RCMs and sequence composition features are important for distinguishing circRNAs from lncRNAs. There are also 2 graph features in the top 10, which means there is a certain effect in terms of distinguishing the two.

Then, a violin plot was drawn to visualize the horizontal vector of the feature selection results and observe the distribution characteristics of the data points (Fig. 3E). According to the scores generated after feature selection, we selected the top-ranked two-dimensional features from the results of each feature expression method and drew violin plots to visualize the data distribution of positive and negative examples (“0” is positive, and “1” is negative). By observing the distribution of data points in Fig. 3E, it was found that the distributions of positive and negative examples of RCMs, conservative scores, graph structures, and composition features are relatively obvious, while the differences in the distributions of positive and negative examples of RCMs features are relatively small. This result shows that the feature representation method has a strong influence on the distributions of positive and negative samples and contributes greatly to the final classification results. This poses a new challenge on how to use more efficient features to improve the performance of classifiers.

### Feature importance analysis and feature contribution and dependency analysis

**Fig. 4:**
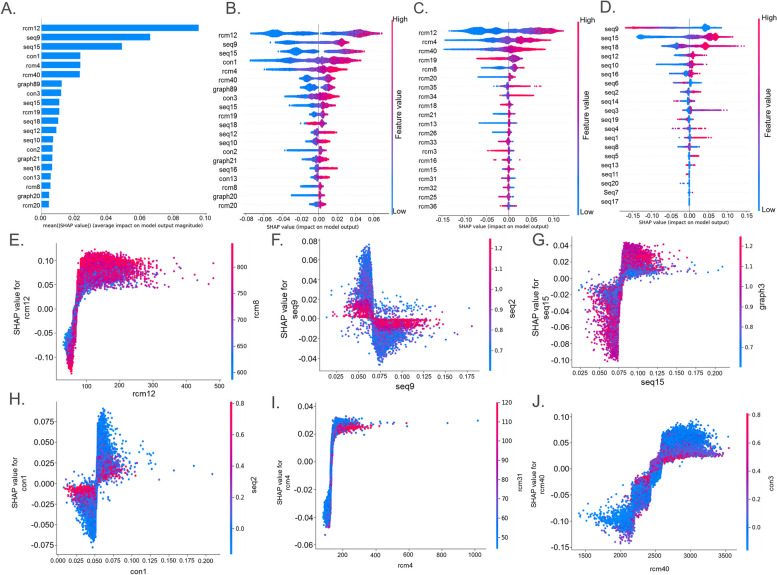
Feature contribution and dependency analysis. **A** The 20 most important features. **B** Summary plot for SHAP values. For each feature, one point corresponds to a single sample. The SHAP value along the *x*-axis represents the impact that feature had on the model’s output for that specific sample. Features in the higher position in the plot indicate the more important it is for the model. **C** Summary plot for SHAP values for RCMs features. **D** Summary plot for SHAP values for graph structure features. **E**–**J** SHAP dependence plots. These plots show the effect that a single feature has on the model predictions and the interaction effects across features. Each point corresponds to an individual sample, the value along the *x*-axis corresponds to the feature value, and the colour represents the value of the interacting feature

We used Shapley additive exPlanations (SHAP) [27] to analyse the feature contributions and dependencies. The SHAP value represents the contribution of a feature to the model output variation, reflects the influence of the feature in each sample, and can also show positive and negative effects. First, the absolute SHAP values of each feature in circRNA were averaged, and the 20 most important features were calculated and described with a SHAP summary map, as shown in Fig. 4A. Of the first 20 dimensions, 6 are RCMs features, 4 are conservative score features, 3 are graph structure features and 7 are sequence features. RCM features and sequence composition features accounted for a large proportion of features; thus, they play an important role in the recognition of circRNA.

Then, to better understand the relationship between the eigenvalues and the model output and thus the pattern of the overall sample characteristics, a summary graph was constructed. The SHAP values of each feature of each sample were counted, and outliers were observed if present. Figure 4B shows a summary plot of the top 20 most important features, and Figs. 4C and D show summary plots of the RCMs and sequence compositional features, respectively. First, on the vertical axis, the features are sorted according to the sum of the SHAP values of all samples, and the horizontal axis is the SHAP value. First, the rcm12 feature is the most important, and the larger the rcm12 value is, the better the model prediction output. The output will decrease significantly once a certain peak is reached. rcm12 will increase the probability that the sample is predicted to be circRNA, that is, this feature has a positive impact on the prediction of circRNA. seq9 is also an important feature that affects the results. The larger the seq9 value is, the better the performance of the model. seq15 and the model performance are roughly negatively correlated. Second, similar results are observed for the other 9 features, such as seq9, which is positively correlated with the predicted value. Seven features, such as rcm40 and graph89, show the opposite result, that is, high eigenvalues will reduce the performance of CircDC, while low eigenvalues will improve the performance of CircDC. rcm12 (− 0.26–0.19) and seq9 (− 0.14–0.16) have large variation ranges and play key roles in the output of CircDC, which also proves that the features dominate the behaviour of the model. These features are followed by seq15, con1 and the change of the eigenvalues of rcm4. rcm20 has less impact on the output performance of the model, and its importance is lower than that of other features. Because the RCMs and sequence composition features account for a relatively large proportion of the first 20-dimensional features, we further analysed the impact of the RCMs and sequence composition features on the model output. The key roles of rcm12, rcm4, rcm40, seq9, seq15, and seq18 on the features were identified.

Then, to understand how a single feature affects the output of the model, we analysed the SHAP values of the top 6 features and compared them with the feature values of all samples in the dataset. To help reveal these interactions, another feature was automatically selected for colouring, and a dependency graph was drawn (Fig. 4E–J). It can be observed that the proposed CircDC is characterized by a turning point when the rcm12 value is approximately 100. Larger rcm8 values reduce the influence of the rcm12 feature when the rcm12 value is 100 but increase the influence when this value is greater than 100. Higher rcm8 values will change the SHAP value from negative to positive, making rcm12 positively correlated with the output of CircDC. The inflection point of seq9 is 0.0, 70. The samples with lower eigenvalues of seq2 will have an impact on seq9, and the samples with higher eigenvalues will have less impact on seq9. The graph structure feature graph3 will interact with the component feature seq15, which will affect the prediction output of the model. The conservative score con1 will interact with the composition feature seq2 and affect the output of the model. High rcm4 values (200–500) and high rcm31 values (100, 120) are helpful for the model to accurately predict circRNA, while low rcm31 sample feature values have the opposite effect. More feature interaction dependence graphs are provided in Additional file 2.

### Expanding the application of our CircDC framework in circRNA identification tasks

**Fig. 5:**
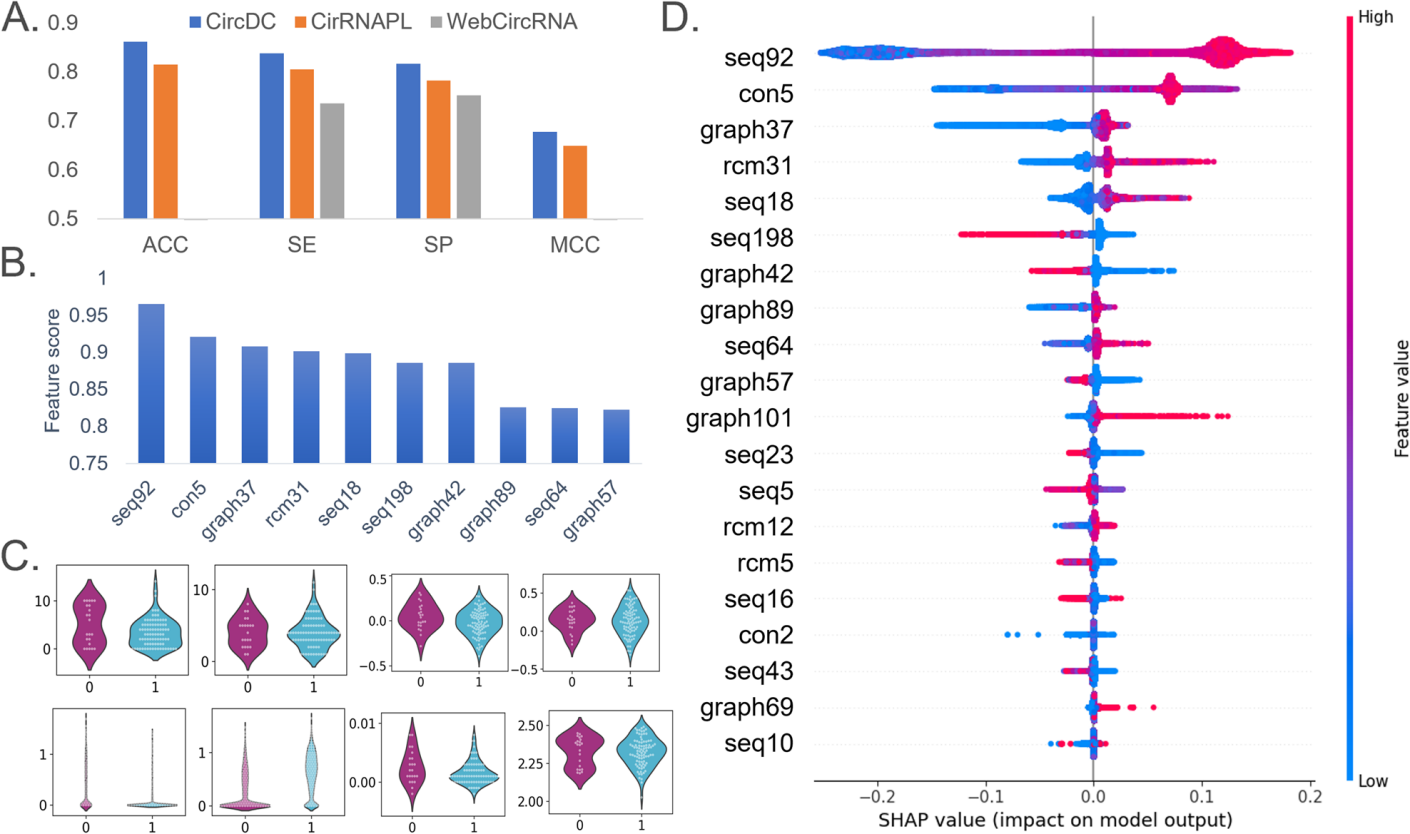
**A** Performance comparison with state-of-the-art methods. **B** Top 20 feature analysis. **C** Violin plot to visualize feature distribution. **D** Summary plot for SHAP values

**Fig. 6:**
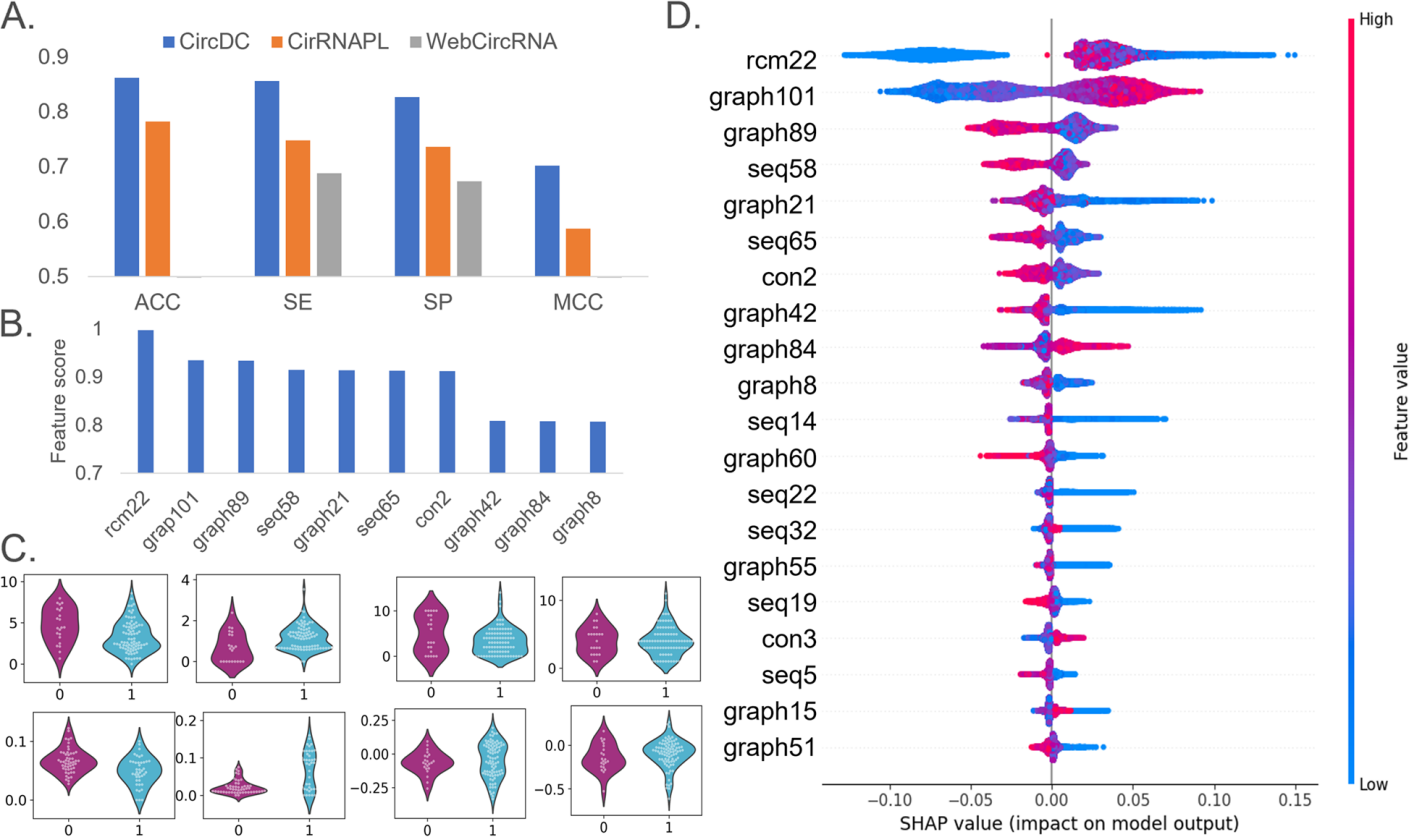
**A** Performance comparison with state-of-the-art methods. **B** Top 20 feature analysis. **C** Violin plot to visualize feature distribution. **D** Summary plot for SHAP values

After building the identification model for circRNA and lncRNA, we expanded and applied our framework. Retrain the model based on the extended application's data set and extend the application. First, many circRNAs are produced by protein-coding genes (PCGs). More than half of the human circRNAs in circBase were derived from PCGs [28]. We collected PCGs that do not overlap with circRNAs and applied this prediction framework to train a model to predict and identify differences between PCGs and circRNAs. Second, circRNAs are expressed in a cell/tissue-specific manner. circRNAs are expressed in stem cells and are particularly prominent during embryonic development [29]. After we collected the circRNA and determined that the sequence was circRNA, we downloaded the data of stem cell expression from circBase and used this model to determine whether the circRNA was expressed in stem cells. And apply the CircDC prediction framework to train the model to predict whether circRNA is expressed in stem cells and explain the difference in characteristics. Figures 5A–D and 6A–D show the prediction results of circRNA and PCGs and the prediction results of whether circRNA is expressed in stem cells, respectively. It can be observed that our CircDC framework achieved higher performance than that of the evaluated method on most metrics when predicting. Moreover, similar results were obtained in stem cell expression prediction.

#### The CircDC architecture is outstanding in the prediction of circRNAs and PCGs

We first downloaded the PCGs data from the GENCODE database, and we also collected 20,345 PCGs from GENCODE v19. To ensure clear binary classification for training, we removed PCGs overlapping circRNAs in circBase, resulting in 9,533 PCGs. Then, the dataset was input into CircDC to identify circRNAs and PCGs, and the performance was compared with that of the WebCircRNA and CirRNAPL models, as shown in Fig. 5A. Using our CircDC framework, the ACC is 0.8614, the SE is 0.8381, the SP is 0.8165, and the MCC is 0.6774, which are 0.1021 and 0.0645 higher than the SE and SP of WebCircRNA, respectively, and 0.0464, 0.0331, 0.0345 and 0.0284 higher than the ACC, SE, SP and MCC of CirRNAPL, respectively. The CircDC framework has good performance in terms of circRNA and PCG prediction and classification and is an improvement over existing methods.

The results of feature selection were then analysed (Fig. 5B). Among the top 10 features, there are 4 sequence composition features, 1 conservative score, 1 RCMs feature, and 4 graph structure features. Sequence composition features and graph structure features are most important for distinguishing circRNAs from PCGs. Then, a violin plot was drawn to visualize the horizontal vector of the feature selection results (Fig. 5C) and observe the distribution characteristics of the data points. The differences in the distributions of positive and negative examples for the RCMs, graph structure, and composition features and conservative score are relatively obvious, while the differences in the distributions of positive and negative examples for RCMs features are relatively small. Finally, an interpretable analysis of the features was performed, as shown in Fig. 5D. It can be found that first, the seq92 feature is the most important, and the larger the value of seq92, the better the model prediction output. This value will decrease significantly once a certain peak value is reached, and it will increase the probability that the sample is predicted to be circRNA, that is, this feature has a positive impact on the prediction of circRNA. Second, graph89 graph37, rcm31 and seq18 are also observed and are positively correlated with the predicted value. Seven features, such as graph198 and graph42, show the opposite result, that is, high eigenvalues will reduce the performance of the model, while low eigenvalues will improve the performance of the model.

#### The CircDC architecture is outstanding in the prediction of circRNA and stem cell expression

Based on circBase, we collected 2082 circRNAs only expressed in H1hsec and randomly selected the same number of circRNAs not expressed in H1hsec from other cell lines. Then, the dataset was input into CircDC to predict whether circRNA is expressed on stem cells and compare the effects with those of the WebCircRNA and CirRNAPL models, as shown in Fig. 6A. The ACC using our CircDC model is 0.862, the SE is 0.856, the SP is 0.827, and the MCC is 0.702, which are 0.168 and 0.154 higher than the SE and SP of WebCircRNA, respectively, and 0.08, 0.108, 0.091 and 0.115 higher than the ACC, SE, SP, and MCC of CirRNAPL, respectively. The CircDC framework has good performance in terms of predicting whether circRNA is expressed in stem cells, and it reflects an improvement over existing methods.

The results of feature selection were then analysed (Fig. 6B). Among the top 10 features, there are 2 sequence structure features, 1 conservation score feature, 1 RCM feature, and 6 graph structure features. Graph structure features are the most important for distinguishing whether circRNAs are expressed in stem cells. Then, a violin plot was drawn to visualize the horizontal vector of the feature selection results (Fig. 6C) and observe the distribution characteristics of the data points. The differences in the distributions of positive and negative examples of graph structure features are relatively obvious, while the differences in the other three are relatively small. Finally, an interpretable analysis of the features was performed, as shown in Fig. 6D. First, the rcm22 feature is the most important, and the larger the rcm22 value is, the better the model prediction output. This value will decrease significantly once a certain peak value is reached. It will increase the probability that the sample is predicted to be circRNA, that is, this feature has a positive impact on the prediction of circRNA. Graph101 is also an important feature that affects the results. The larger the graph101 value is, the better the performance of the model. Many features, such as graph89, seq58, and graph21, are negatively correlated with the model performance. The larger the feature value is, the worse the model performance.

## Conclusions

Although a number of computational models have emerged for circRNA prediction, their identification accuracy still needs to be improved, and the looping characteristics of circRNAs are insufficient. This paper constructs a new circRNA classifier CircDC. Compared with existing methods, our method has the following advantages: (i) Introducing a new feature extraction RCM, which can provide the possibility of cyclization for a given flanking sequence and query sequence. (ii) Using CNN and BLSTM for deep feature learning and circRNA prediction, the accuracy increased by more than 3%. (iii) Through feature importance analysis, we also found that RCM is used to distinguish circRNAs and lncRNAs. It can help researchers extract useful information from a large amount of biological data to reveal the biological processes and mechanisms of organisms. (iv) Extend CircDC to differentiate between circRNA and PCG, and whether circRNA is expressed in stem cells, and analyze the importance of features.

Future work will focus on discovering more useful features and combining parallel techniques to improve recognition efficiency. In addition, there are currently few studies on the time complexity and space complexity of deep learning frameworks. In the next step, we will also analyze the efficiency of building a deep learning framework from more perspectives. At the same time, given that the functions of most circRNAs have not been confirmed by research, and the functions of disease-related circRNAs still need to be continuously improved, future research should pay more attention to the biological functions of circRNAs.

## Methods

### Dataset collection

To demonstrate the capability of our CircDC model, we used circRNA data from the circBase [30] and circRNADb [31] databases. The circBase and circRNADb databases collect experimentally validated multispecies circRNA transcript data. We downloaded all human circRNA data and deleted sequences with sequence lengths < 200 nt. Next, CD-HIT was used to remove redundant data, and the similarity threshold was set to 0.8. Then, 14,480 human circRNA sequences were obtained from circBase, and 31,939 sequences were obtained from the circRNAdDb. By comparing the circRNA IDs of the circBase and circRNADb, it was found that the circRNADb data cover the circBase data. If data overlap between the two databases is allowed, 32,571 sequences can be obtained, including 9848 repeated sequences and 26,723 unique sequences. We used GENCODE to collect experimentally validated, human-annotated lncRNA sequences and obtained 19,683 sequences as negative samples.

This paper uses TFCV to verify the effectiveness of the model. Among them, the number of positive samples is 32,571. The number of negative samples is 19,683. For the positive samples in the independent validation set test, we used the 9848 repeated sequences as the test set and the 26,723 unique sequences as the training set. Negative samples were directly divided into training and test data, of which 80% were used for training and 20% were used for testing.

### Feature representation

#### RCMs

Inverse complement match-mediated RNA circularization is a classical strategy for circRNA overexpression. Inverse complement matching of circRNA flanking introns is highly correlated with circularization [32]. When RCMs occur upstream and downstream of exon 2, a circular molecule is formed; otherwise, a linear molecule is formed. RCMs between introns encircling the circRNA may induce larger hairpin structures, thereby facilitating changes in loops embedded in exons. Therefore, we hypothesized that strengthening the hairpin between reverse complement sequences might increase the probability of circularization, and the longest reverse complement sequence in the flanking sequence was measured to represent the absolute number of all reverse complement sequences in the flanking sequence. In this paper, RCMs were characterized by calculating the strength of hairpins in the flanking sequences (fraction H of the reverse complements), where the presence or absence of reverse complements in the flanking sequences was calculated by deriving the H value, which represents the fraction of all reverse complements in the flanking sequences.

First, the reverse complementary sequence was introduced. The reverse complementary sequence reverses the original sequence first and then complements the reverse sequence. The concept of complementarity refers to the A-T and C-G pairings. For example, if the original sequence is AATTCCGG, then the reverse sequence is GGCCTTAA, and the complement of the reverse sequence is CCGGAATT.

The reverse complement score H of the sequence was then calculated. For sequence C, two flanking sequences of length L base pairs are selected. Then, a sliding window method was used to split the flanking sequences into k-mers, all subsequences of length k and stride s = 1 were extracted, and L1 = L0-k + 1 subsequences with D = 4^ k^ possible words of length k in the sequence were obtained. For W_i_ $({\text{i}}=1,\cdots ,{4}^{k}$), the reverse complement sequence is W_R_. Then, the number of occurrences of the reverse complement sequence in the left flanking sequence N(W_i_) and the number of times it appears in the right flank sequence N(W_R_) were counted. The reverse complement score H $(k,{L}_{0}$) was calculated as Formula (1).

1 $$H\left(k,{L}_{0}\right)=\sum_{i=1}^{{4}^{k}}{H}_{i}$$

2 $${H}_{i}={\text{min}}\left(N\left({w}_{i}\right),N\left({w}_{R}\right)\right)$$

where *W*_*R*_ is the reverse complement of *W*_*i*_. For example, *W*_*R*_(ACCGU) = ACGGU.

#### Conservative score features

Conservation scoring is an evolutionary concept, and the sequence of the genome is constantly evolving. Many circRNAs have not disappeared in the long evolution and are conserved in species, indicating that their functions are not accidental [33]. Therefore, we analysed the conservation of circRNA by calculating the conservation score and performed more accurate feature expression.

For each sequence, we used the PhastCons [34] method to collect precalculated conservation scores from the UCSC database and score each nucleotide based on its degree of conservation. The phyloP conservation score for each base and the mean and standard deviation of the conservation score length for the entire sequence were calculated. First, we averaged the scores for each exon sequence in each transcript and then calculated the maximum, mean and median of the averaged scores. Therefore, the frequency numbers of consecutive bases with scores greater than the specified threshold were summed, and the frequency numbers were then divided by the entire length of the sequence to obtain a total of 15 features to create the conserved descriptor.

#### Graph structure features

RNA-binding proteins can participate in the regulation of the formation of related circRNAs in epithelial-mesenchymal cells by binding to precursor RNA flanking introns and by adding protein-binding sites to linear RNA flanking introns to facilitate the formation of aggregates [35]. In addition, these proteins can also play an important role in various life activities in the form of RNA‒protein copolymers by selectively combining with circRNA. Therefore, we used the GraphProt method with an efficient graph kernel to learn the sequence and structure binding preferences of RBPs and to calculate the graph structure features of sequences.

For each sequence, a set of RNA structural features was generated using GraphProt [36]. The basic process is described below (shown in Fig. 1B(3)). First, the secondary structure was analysed using the stem (S), polyloop (M), hairpin (H), internal loop (I), bulge (B), and external region in RNAshapes. Then, the circRNA sequence and its folded structure were encoded as a graph, using the graph form to represent different types of relationships. The RNA graph uses nodes to represent nucleotides and edges to represent the sequence backbone connection or the bonding relationship between base pairs. For example, in structural base pairs, the nucleotide C inside the stem can be labelled CS and CB inside the raised loop. Finally, the graph was feature-encoded based on the neighbourhood subgraph pairwise distance kernel. The graph was decomposed into a set of small overlapping subgraphs. We then used a hash-based technique to assign a numerical identifier to each subgraph to approximate the isomorphism detection problem and construct the final explicit feature encoding. Then, we obtained a set of over 30,000 graph features. To reduce the dimensionality of graph-structured features, we used an RF to rank the importance scores of the graph features, resulting in 101 graph features with high importance scores.

#### Sequence composition features

With the help of the iLearn package [37], we investigated the composition characteristics of circRNA sequences, where the sequence composition characteristics include nucleic acid composition characteristics (including k-mer, mismatch, and subsequence), autocorrelation characteristics (including DAC, DCC, MAC and GAC) and pseudoribonucleic acid composition.

### Feature selection

In this paper, the MRMD 2.0 [25, 38] was used for feature selection. The MRMD 2.0 first calculates the feature selection results of seven feature sorting algorithms (ANOVA, MRMD, MIC, LASSO, MRMR, chi-square, recursive feature elimination) and builds a directed graph in the form of a linked list with the results of the seven methods. Then, the PageRank algorithm is used to sort the linked list to obtain the final feature set.

### Deep learning model

The CircDC model constructed in this paper is mainly composed of a CNN [39] and BLSTM [40, 41] network. TensorFlow v2.4 is used to implement the deep learning model [42]. The CNN consists of convolutional layers. The obtained four sets of features are input into the convolution layers, and new deep features are extracted from the original sequence features. The number of convolution kernels in each convolutional layer is the same, that is, the output feature dimensions are the same. Convolution is used to further mine the relationships between different features, strengthening the connections between extracted features and improving the accuracy of the prediction results. Pooling layers are used to reduce the data dimensionality. A BN layer is used to normalize the data to speed up the training process. Then, the obtained features are concatenated into a vector, which is input into the BLSTM network to extract latent feature patterns and capture the short-term and long-term sequential dependencies between features. The output of the BLSTM unit in the last layer serves as the input to three fully connected layers. To reduce the overfitting of the model, a dropout layer is added. The ReLU function is used in the two fully connected layers, and the softmax function is used in the final output layer for binary classification.

### Performance metrics

We used four indicators to evaluate the performance of our CircDC model, namely, SE, SP, ACC and MCC [43]. SE represents the correct rate of positive sequence prediction. SP represents the correct rate of negative example prediction. ACC stands for classification accuracy. The MCC reflects the reliability of the classifier and can more fairly reflect the predictive ability. The larger the MCC is, the better the reliability.

### Supplementary Information

**Additional file 1: Table S1.** The final optimal architecture and hyperparameters.

**Additional file 2: Figure S1.** The SHAP dependence plots.

## Data Availability

All code and data generated or analyzed during this study are included in this published article, its additional file, and publicly available repositories. Which are available in the Zenodo repository (https://zenodo.org/record/8186032) and GitHub (https://github.com/nmt315320/CircDC.git).

## Abbreviations

circRNAs: Circular RNAs
ML: Machine learning
RF: Random forest
ELM: Extreme learning machine
DL: Deep learning
RCMs: Reverse complementarity matching features
MRMD: Max-relevance-max-distance
CNN: Convolutional neural network
BLSTM: Bi-directional Long Short-Term Memory
TFCV: Tenfold cross-validation
ACC: Accuracy
LR: Learning rate
SE: Sensitivity
SP: Specificity
ACC: Accuracy
MCC: Matthews correlation coefficient
ROC: Receiver operating characteristic curve
SHAP: Shapley additive exPlanations
PCGs: Protein-coding genes
