# Supplementary material for: CircRNA identification and feature interpretability analysis
Source: BMC Biol. 2024 Feb 27;22:44. doi: 10.1186/s12915-023-01804-x (PMC10898045; doi:10.1186/s12915-023-01804-x)
Supplement: Supplementary file 1 — Additional file 1: Table S1. The final optimal architecture and hyperparameters. [file 12915_2023_1804_MOESM1_ESM.docx]

Table S1. The final optimal architecture and hyperparameters

| Parameter | Value | Parameter | Value |
| --- | --- | --- | --- |
| Epoch | 200 | Pool kernel size | 4 |
| Learning rate | 0.001 | convolutional kernel size | 3 |
| decay_rate | 0.0005 | batch | 64 |
