# Supplementary figures and images for: CircRNA identification and feature interpretability analysis
Source: BMC Biol. 2024 Feb 27;22:44. doi: 10.1186/s12915-023-01804-x (PMC10898045; doi:10.1186/s12915-023-01804-x)

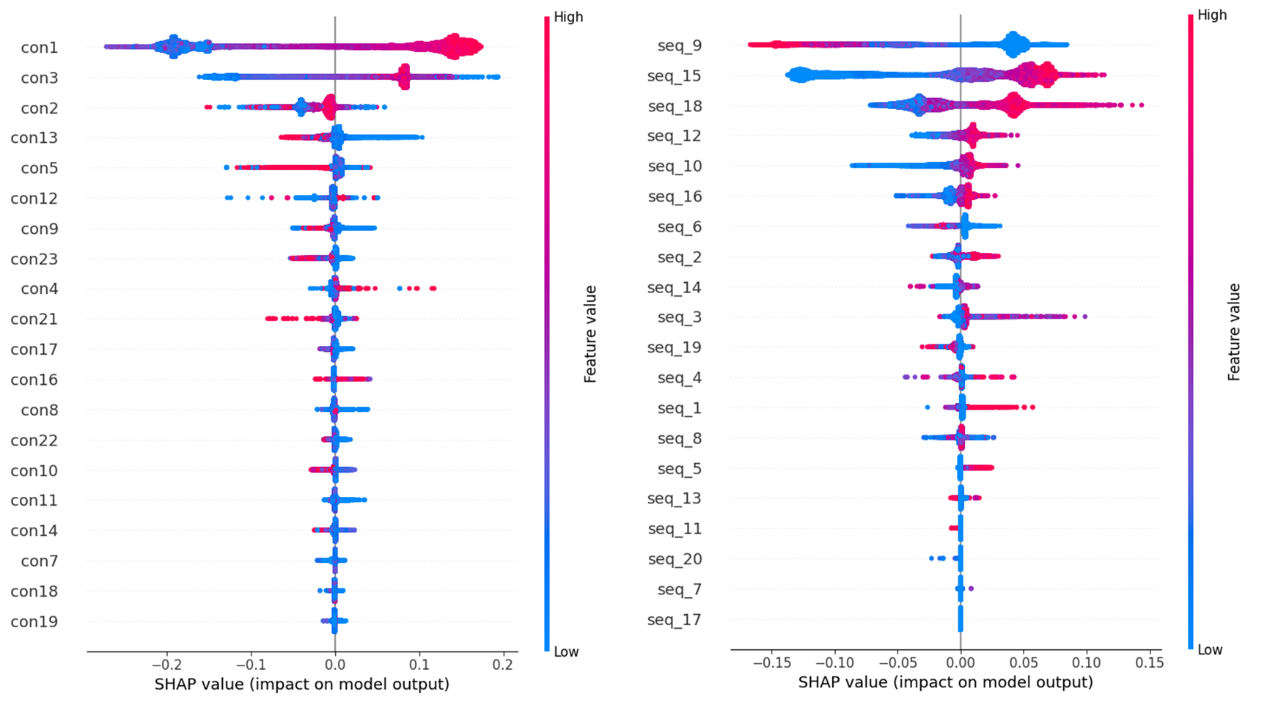


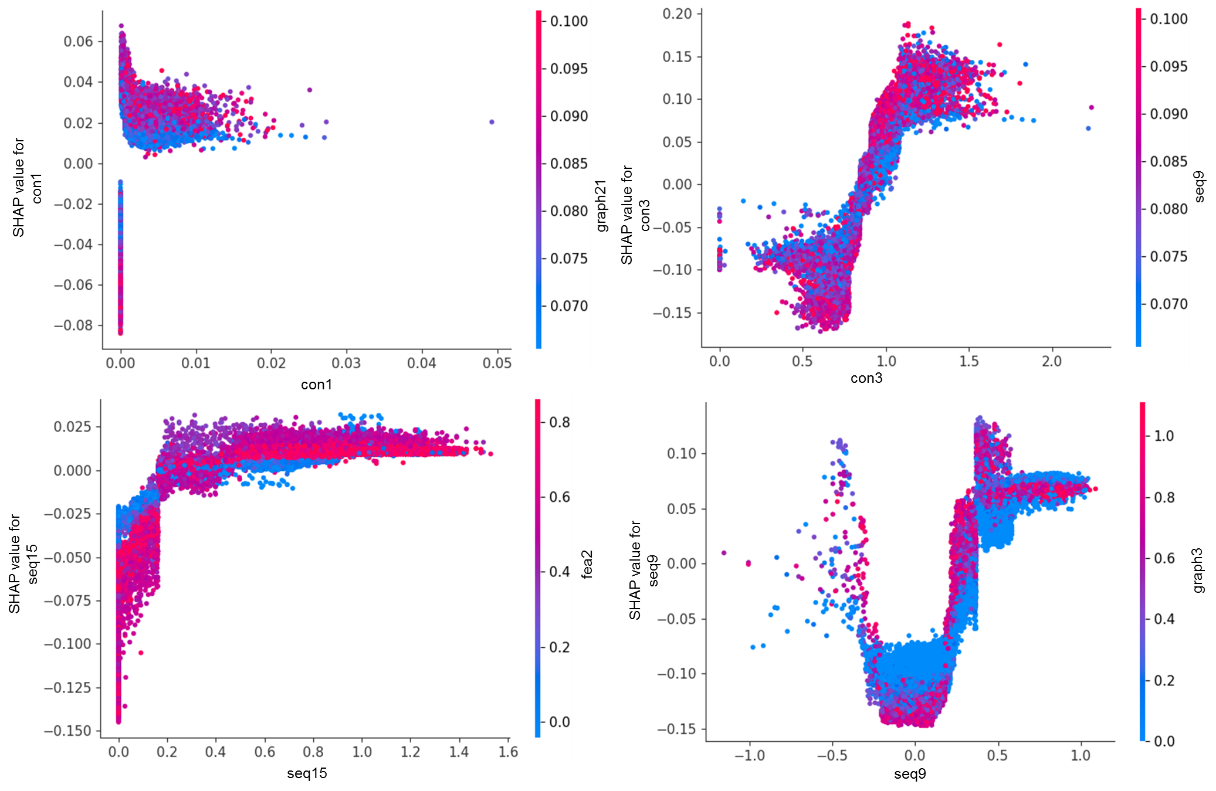


Figure S1. The SHAP dependence plots.

Supplement: Supplementary file 2 — Additional file 2: Figure S1. The SHAP dependence plots. [file 12915_2023_1804_MOESM2_ESM.docx]
